# Influence of Grinding Parameters on Surface Roughness and Subsurface Crack Damage Depth of Sapphire Crystal

**DOI:** 10.3390/ma18112461

**Published:** 2025-05-24

**Authors:** Yingqi Hou, Yufei Gao, Chunfeng Yang

**Affiliations:** 1 Key Laboratory of High Efficiency and Clean Mechanical Manufacture of MOE, School of Mechanical Engineering, Shandong University, Jinan 250061, China; 2 State Key Laboratory of Advanced Equipment and Technology for Metal Forming, Shandong University, Jinan 250061, China; 3 Shandong Key Laboratory of High Performance Tools and System, Shandong University, Jinan 250061, China

**Keywords:** sapphire crystal, grinding, surface roughness, subsurface crack damage, grinding parameters

## Abstract

Single sapphire crystals has been widely used in technology such as light emitting diodes, lasers, high-temperature and high-voltage devices, special windows, and optical systems, and grinding is an important process of their machining. In order to reveal the influence of the grinding wheel speed, grinding depth, and feed rate on the ground surface quality of sapphire crystals, a three-factor and five-level orthogonal experiment was designed and completed. Variance and range analysis was conducted on the experimental results using the surface roughness *Ra* and subsurface crack damage depth (SSD) as evaluation indicators, and optimized parameter combinations were explored. Furthermore, mathematical prediction models for the power regression of the *Ra* and SSD were established based on the experimental data. The research results indicate that within the range of the process parameters used in this experiment, the grinding process did not achieve the full ductile removal of the material. Some of the material was removed in a brittle mode, forming fractured pits on the ground surface, and median crack propagation occurred in the subsurface, forming a subsurface microcrack damage layer. The influence of the grinding parameters on the *Ra* and SSD showed a consistent trend, which was that the parameter with the greatest impact was the grinding wheel speed, followed by the feed rate and grinding depth. The *Ra* and SSD obtained under the optimized grinding parameter combination were 0.326 μm and 2.86 μm, respectively. The research results provide an experimental basis and guidance for improving the surface quality of sapphire crystals during grinding.

## 1. Introduction

Single sapphire crystals have the advantages of extremely high hardness and structural strength, wear resistance, a low material density, and the ability to withstand ultra-high-temperature conditions. Therefore, they are widely used in technology such as light emitting diodes (LED) [1], lasers [2], high-temperature and -voltage devices [3], special windows, and optical systems [4]. At present, sapphire crystals are generally cut using a diamond wire saw [5,6,7,8,9], and some of the subsequent precision and ultra-precision machining processes are grinding and polishing. Grinding is an important machining process that occurs between cutting and ultra precision polishing. There are microcracks and saw marks on the surface of sapphire wafers cut using diamond wire saws [6,7], which need to be eliminated during grinding to improve the flatness of the wafers. It is also hoped that the depth of the microcrack damage in the subsurface of sapphire wafers cut using a diamond wire saw will be reduced through the grinding process [8,9]. After the grinding process, the wafer needs to be polished to remove surface defects formed during the grinding process, such as brittle pits, scratches, and subsurface microcrack damage layers. Therefore, the surface and subsurface quality resulting from grinding has a significant impact on the subsequent polishing processing time, directly affecting the processing cost.

In sapphire grinding processes, the quality of the surface and subsurface is a widely discussed issue among scholars. Scholars have conducted extensive research on the ground surface’s morphology and formation mechanism, surface roughness, and subsurface microcrack depth. In terms of process parameters that affect the machining quality, the main focus has been on the grinding wheel speed, grinding depth, and feed rate. The ground surface’s roughness and subsurface microcrack damage need to be remedied through subsequent polishing processes to ultimately obtain a surface without crack damage, with the surface roughness reaching several nanometers [10,11]. Yang et al. [12] observed the surface morphology of ground sapphire and found that under the brittle fracture material removal mode, the surface material of sapphire is prone to peeling off and forming irregular pits, resulting in a surface roughness of between 0.33 and 1 μm. A higher grinding wheel speed is not conducive to obtaining a low surface roughness and the greater the grinding depth, the more significant the cleavage fracture characteristics of the machined surface. Wang et al. [13] found in their study on the surface morphology of ultra-precision sapphire grinding that controlling the grain size of the grinding wheel and reducing the grinding depth can enable ductile material removal during grinding, and the surface roughness *Sa* can reach below 0.1 μm. Wang et al. [14] monitored the grinding signals and surface quality of sapphire in ultra-precision grinding on different crystal faces and characterized the roughness and surface damage. Research has found that surface roughness and damage characteristics have no significant effect on the ductility of material removal but have a significant impact on the brittleness of material removal. Hu et al. [15] studied and compared the grinding characteristics of the different crystal faces of sapphire. Their research results showed that the fracture toughness of the C-plane was the highest, and it had a certain inhibitory effect on the propagation of cracks during grinding and material removal, resulting in less brittle fractures in the surface of the C- plane. Cheng et al. [16] found in their experimental study that the size of the surface cracks in sapphire crystals during grinding increases with an increase in the feed rate and decreases with an increase in the grinding wheel speed. A fractal analysis of the ground surface of sapphire crystals also revealed that changing the process parameters will affect the brittleness or ductility of the material removal mode [17]. When comparing the down grinding and up grinding methods under the same process parameters, it was found that the down grinding method resulted in fewer micro pits on the ground surface and better quality [18,19].

The depth of subsurface microcrack damage is another important issue in the grinding process for sapphire crystals. A low depth of microcrack propagation will greatly reduce the subsequent polishing processing time. Wang et al. [13] found that the damage to the ground subsurface of sapphire was dominated by median cracks with depths ranging from 8 to 20 μm. Even for ground surfaces with ductile material removal, subsurface microcrack damage cannot be completely avoided. Other than the grinding wheel parameters, the grinding process parameters are important factors affecting the subsurface damage caused to sapphire crystals during grinding [20]. Ultrasonic vibration-assisted grinding can reduce the depth of subsurface crack damage on the ground surface of sapphire crystals [21], but this process is not widely used in industry. Therefore, the selection of appropriate processing parameters is essential for achieving a high-quality ground surface. Xiao et al. [22] proposed a prediction method for the grinding parameters (grinding depth, feed rate, and wheel speed) to achieve a specific surface roughness and subsurface damage depth. This method was based on using experimental data from grinding to establish a genetic algorithm backpropagation neural network model for determining the relationship between the grinding parameters and surface integrity parameters. In other cases, a design of experiments (DoE) approach has been widely used to analyze the influence laws and degrees of the process parameters, optimize the process parameters, and establish regression models. Wasmer et al. [23] applied this method to analyze the influence of five processing factors, the wheel speed, feed speed, vertical feed rate, presence of ultrasonic assistance, and crystallographic direction, on the quality of sapphires subjected to grinding. The research suggested that the wheel speed, feed speed, and vertical feed rate have the most significant impact on the grinding quality and the grinding process parameters were further optimized based on the experimental data. The DoE approach is also widely used in the analysis and optimization of the process parameters for other machining methods, such as diamond wire saw cutting [24], milling [25], and polishing [26].

According to the research results obtained by scholars, the process parameters have a significant impact on the surface and subsurface quality of sapphire crystals during grinding. It is necessary to conduct systematic experimental research, which is of great significance for improving the quality of grinding and reducing the cost of polishing processes. In this paper, an orthogonal experiment on grinding sapphire crystals was designed, using three factors, the grinding wheel speed, grinding depth, and feed rate, and five levels were selected for each factor. Range and variance analysis was conducted on the experimental results to determine the influence of these three factors on the surface roughness and subsurface microcrack damage resulting from grinding and to determine the optimal process parameters. Furthermore, a regression prediction model for the surface roughness and subsurface crack damage depth was established based on the experimental results. This paper systematically analyzed the influence of grinding process parameters on the surface quality of processed sapphire crystals using the DoE method, and the research results provide an experimental basis for improving the quality of sapphires subjected to grinding and optimizing the process parameters.

## 2. Materials and Methods

### 2.1. Experimental Material and Equipment

**Figure 1:**
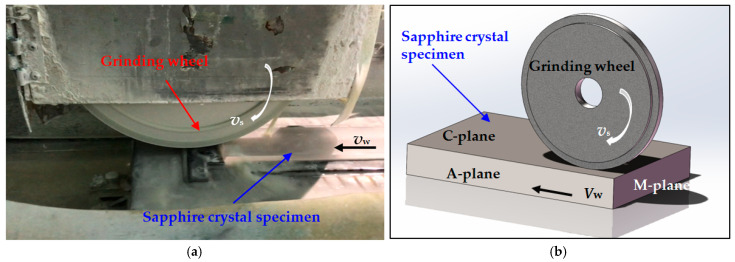
Appearance (**a**) and schematic diagram (**b**) of grinding process.

The grinding experiments were conducted on a precision surface grinder (HM60AHR, Hangzhou Hangmo CNC Machine Tool Co., Ltd., Hangzhou, China). The appearance and a schematic diagram of the grinding process are shown in Figure 1. When grinding materials with high hardness, using the down grinding method can improve the machining accuracy. Therefore, this experiment adopted a wet down grinding process. The grinding wheel rotated at a speed of *v*_s_, and the worktable fed the sapphire crystal specimen in at a speed of *v*_w_. A water-based coolant was supplied to the grinding area through a nozzle. A resin-bonded diamond cylindrical grinding wheel with a 20 mm width, a 400 mm outer diameter, and a 20 mm inner diameter was used, and the average abrasive size was 15 μm. Before the experiment, the diamond grinding wheel was first trimmed with a silicon carbide roller and then sharpened with an oilstone. The experiment used a rectangular sapphire crystal specimen (provided by Jiangsu Tianjing Intelligent Equipment Co., Ltd., Xuzhou, China) with a ground surface size of 60 mm × 260 mm and a thickness of 5 mm. The C-plane (0001) of the sapphire crystal was ground in the experiment.

### 2.2. Experimental Design

The grinding process parameters directly affect the ground surface and subsurface quality of the workpiece. In this experiment, the grinding wheel speed (*v*_s_), grinding depth (*a*_p_), and feed rate (*v*_w_) were selected as three factors used to investigate the effects of different grinding parameters on the surface and subsurface characteristics of a sapphire crystal specimen. Five levels were assigned for each factor, and then a three-factor and five-level orthogonal experiment was designed. Table 1 shows the experimental factors and levels of the grinding parameters, with the determination of the five levels for each parameter taking into account the performance of the grinder, and Table 2 shows the parameter combination design for the orthogonal experiment. The surface roughness *Ra* and subsurface microcrack damage depth (SSD) were used as evaluation indicators to characterize the ground surface and subsurface characteristics. Minitab Statistical Software version 2020 (State College, PA, USA) was used for analyzing the ranges and variances of various factors influencing the Ra and SSD values, based on the experimental results.

### 2.3. Evaluation of Ra and SSD of Ground Sapphire Crystal

**Figure 2:**
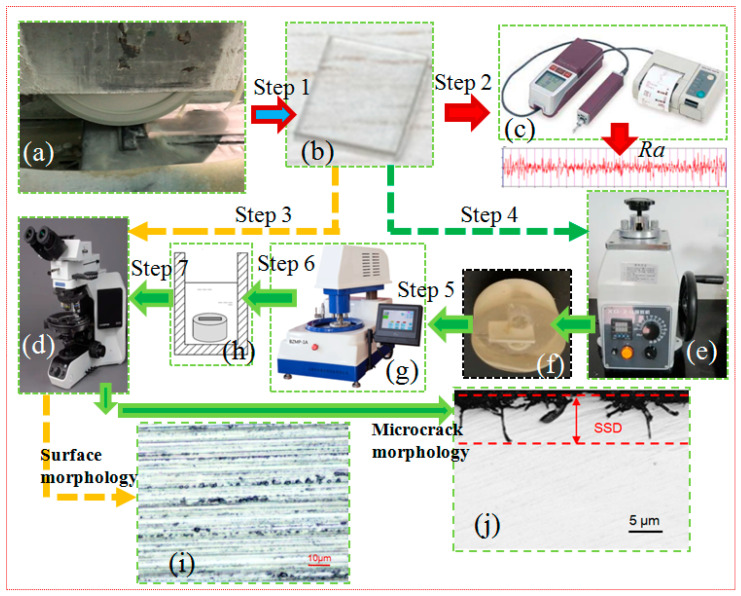
Experimental observation of process of grinding: (**a**) grinding device, (**b**) sapphire wafer, (**c**) surface roughness measuring instrument, (**d**) Olympus optical microscope, (**e**) specimen inlaying device, (**f**) inlayed sapphire wafer, (**g**) lapping and polishing machine, (**h**) corrosion process, (**i**) ground surface morphology, and (**j**) ground subsurface microcrack morphology. Step 1—sapphire wafer grinding process; step 2—surface roughness measurement; step 3—ground surface morphology observation; step 4—inlaying; step 5—lapping and polishing; step 6—corrosion; step 7—SSD measurement.

The surface roughness, *Ra*, and SSD values of the specimens were selected to evaluate the ground surface and subsurface quality. The larger the *Ra* and SSD values of the ground surface and subsurface, the longer the time required for subsurface treatment in the later stage of the polishing process. The ground surface roughness *Ra* was measured by using a roughness measuring instrument, the SJ-201P (Mitutoyo Corporation, Kawasaki, Japan). This measuring instrument uses a contact probe for measurement, with a probe tip angle of 60°. During testing, a measurement length of 2.5mm was taken and a Gaussian Filter was used. Based on the sample length, the *Ra* is the arithmetic mean of the absolute distance between a point on a contour line along the measurement direction and a reference line and is the most widely used surface roughness evaluation parameter in machining. An Olympus BX53M optical microscope (Olympus Corporation, Tokyo, Japan) was employed to observe the ground surface morphology and SSD. The process and details of the experimental observation are shown in Figure 2.

After the grinding experiment was completed, the sapphire crystal specimens were cut into small pieces for observation, as shown in Figure 2b. All specimens were cleaned using an ultrasonic cleaner (Skymen Shenzhen Jiemeng Technology Co., Shenzhen, China). Then, the ground surface roughness and surface morphology were measured and observed separately, shown as step 2 and step 3 in Figure 2, by using the Olympus optical microscope as shown in Figure 2d. Next, an inlaying device was used to inlay the wafer to measure the SSD, as shown in Figure 2e. After lapping and polishing (step 5, as shown in Figure 2g) the thickest cross-section of the sapphire wafer, microcracks in the subsurface layer were exposed by placing it in a corrosive solution (molten KOH at 290 °C) in step 6, allowing for SSD measurement in step 7. The microcracks’ topography and propagation depth in the cross-section of the ground sapphire wafer were also observed and measured by using the Olympus optical microscope, and the depth of the microcrack propagation was characterized as the SSD value. For each specimen, five measurement points were selected in the middle of the stable ground area, and the average value was taken as the measurement result for the *Ra* and SSD.

## 3. Results and Discussion

### 3.1. Effect of Grinding Parameters on Surface Roughness

**Figure 3:**
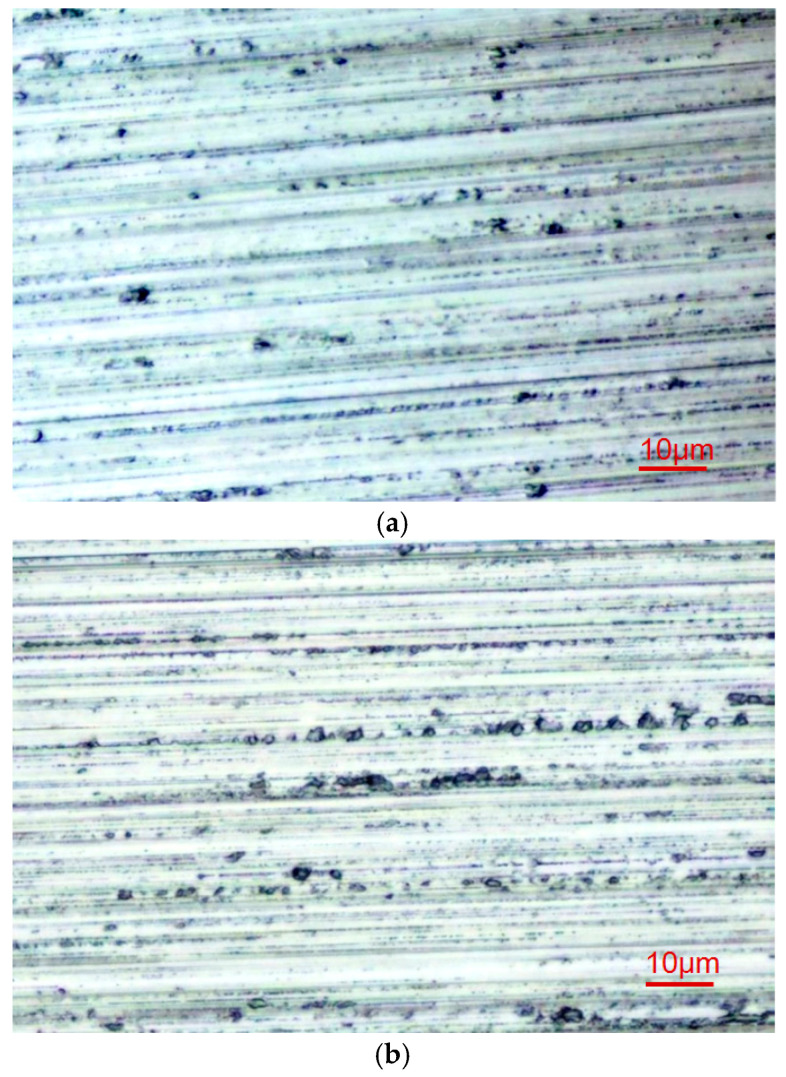
Surface topography of ground sapphire crystal: (**a**) grinding wheel speed of *v*_s_ = 30 m·s^−1^, grinding depth of *a*_p_ = 10 μm, and feed rate of *v*_w_ = 400 mm·min^−1^, and (**b**) grinding wheel speed of *v*_s_ = 15 m·s^−1^, grinding depth of *a*_p_ = 20 μm, and feed rate of *v*_w_ = 1000 mm·min^−1^.

Figure 3 shows the surface microstructure characteristics of the ground sapphire wafers under two different combinations of grinding parameters. As shown in Figure 3, within the range of the grinding parameters in this experiment, the ground surface of the sapphire crystal mainly exhibited smooth grooves formed through ductile material removal, but there were still fracture pits formed through the brittle removal of material, which significantly affected the surface roughness and SSD. Brittle material removal left microcracks in the subsurface layer of the specimen, causing SSD. Comparing the ground surface morphology resulting from the use of different modes shown in Figure 3a,b, it can be observed that it was influenced by different parameter combinations, such as changes in the number and size of the brittle pits, indicating that the grinding parameters had a significant impact on ground surface characteristics such as the *Ra* and SSD.

Table 3 shows the surface roughness, *Ra*, values of the ground sapphire crystal under 25 different grinding parameter combinations determined in the orthogonal experimental design, and the *Ra* value was between 0.35 and 0.95 μm. Table 4 shows the variance analysis results for the *Ra*. The *p*-value represents the significance level of the difference. The *p*-values of the grinding wheel speed, grinding depth, and feed rate were all less than 0.001, indicating that these three process parameters had a significant impact on the roughness of the ground sapphire crystals. Furthermore, a model *R*^2^ = 0.981 indicates that 98.1% of the variation in the surface roughness of the ground sapphire was influenced by these three process parameters. These three parameters were closely related to the material removal rate of the ground sapphire, directly affecting the material removal mode and affecting the surface roughness. The *F*-value reflects the relative degree of influence of each factor, such as the grinding wheel speed, on the response variable, and the larger the *F*-value, the stronger the influence of that factor. According to the *F*-values shown in Table 4, the degree of the influence of each grinding parameter on the surface roughness decreased in the following order: the grinding wheel speed, the feed rate, and the grinding depth.

Table 5 shows the range analysis results for the *Ra*. The corresponding *R* values for the grinding wheel speed, grinding depth, and feed rate were 0.3928, 0.1378, and 0.2622, respectively, which indicates that within the range of the grinding parameters and under the experimental conditions in this paper, the effect of the grinding wheel speed on the *Ra* of the ground sapphire crystal was more significant than that of the other two parameters. In relation to the effects of the other parameters, in this experiment, the effect of the grinding depth on the *Ra* was the smallest. The results of the range analysis were consistent with those of the variance analysis.

**Figure 4:**
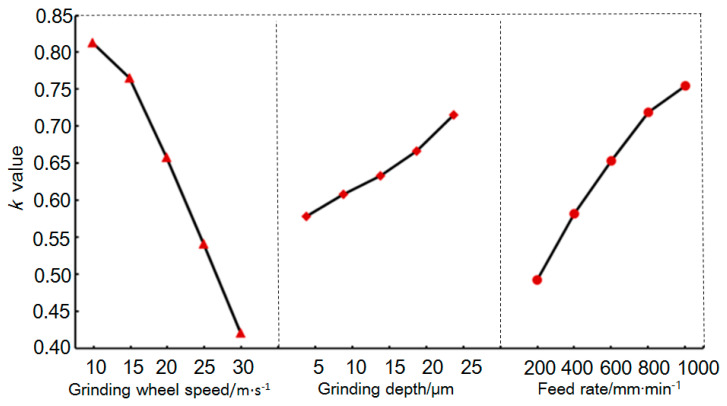
Mean values for various levels of various factors influencing *Ra*.

Figure 4 shows the average values of the influence at different levels of the three grinding parameters on the surface roughness *Ra*. The *K* value on the vertical axis is the average of the experimental results at different levels of each parameter in the orthogonal experiment. Within the range of values of the various grinding parameters studied in this experiment, the surface roughness, *Ra*, value improved with an increase in the grinding wheel speed and a decrease in the grinding depth and feed rate. Increasing the grinding wheel speed increased the number of abrasives passing over the ground surface per unit time, thereby reducing the cutting marks from individual abrasives and lowering the surface roughness. Decreasing the grinding depth and feed rate lowered the grinding force, thereby reducing the brittle fractures in the material in the grinding zone and improving the surface roughness. According to the mean value variation law shown in Figure 4, it can be concluded that the optimal process parameter combination for achieving the minimum grinding surface roughness *Ra* was A_1_B_5_C_1_, that is, a grinding wheel speed of *v*_s_ = 30 /m·s^−1^, grinding depth of *a*_p_ = 5 μm, and feed rate of *v*_w_ = 200 mm·min^−1^.

Based on the orthogonal experimental parameters and results, a power regression analysis was conducted to establish the relationship between the grinding parameters and *Ra*, and a regression prediction mathematical model for the *Ra* was established by using MATLA Version 2020 (Natick, MA, USA).

*Ra* = 0457*v*_s_^−0.582^*a*_p_^0.114^*v*_w_^0.272^ (1)

The index of *v*_s_ is negative, indicating that increasing the speed of the grinding wheel can reduce the ground surface roughness. In Equation (1), the absolute value of each grinding parameter index represents the degree of the influence of that parameter on the *Ra*. The impact of the grinding depth *a*_p_ is minimal.

### 3.2. Effect of Grinding Parameters on SSD

**Figure 5:**
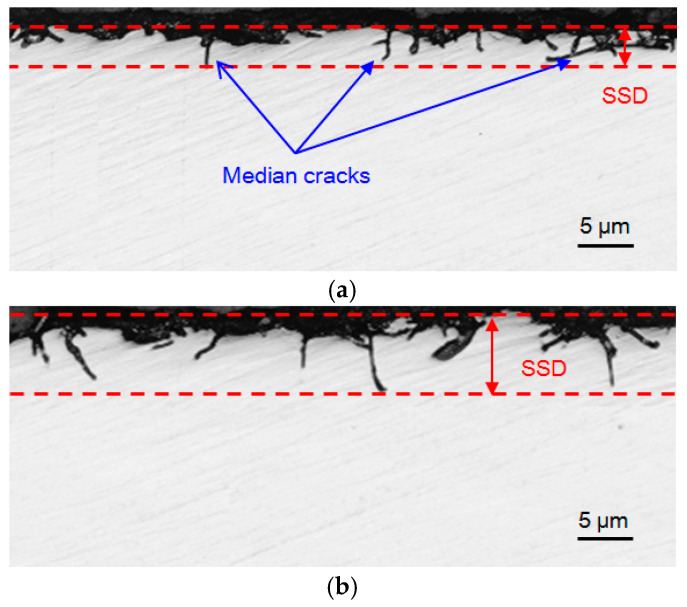
Subsurface microcrack damage to ground sapphire crystal: (**a**) grinding wheel speed of *v*_s_ = 30 m·s^−1^, grinding depth of *a*_p_ = 10 μm, and feed rate of *v*_w_ = 400 mm·min^−1^, and (**b**) grinding wheel speed of *v*_s_ = 15 m·s^−1^, grinding depth of *a*_p_ = 20 μm, and feed rate of *v*_w_ = 1000 mm·min^−1^.

Figure 5 shows the SSD morphology of the ground sapphire wafers under two different combinations of grinding parameters. Median microcracks propagated into the ground subsurface layer, causing subsurface damage. Comparing the microcrack characteristics shown in Figure 5a,b, it can be observed that they were influenced by different parameter combinations, such as changes in the propagation depth and distribution density of microcracks, indicating that the grinding parameters had a significant impact on the ground SSD. Based on the morphology of the ground surface shown in Figure 3, some residual microcracks were exhibited in the subsurface layer when material was removed in the brittle mode. To reduce the time and cost of the subsequent lapping and polishing processes, it is necessary to lower the amount of SSD as much as possible.

Table 6 shows the subsurface crack damage depth, SSD, values of the ground sapphire crystal under 25 different grinding parameter combinations based on the orthogonal experimental design, and the SSD value was between 3.05 and 11.25 μm, which reveals that the SSD had a relatively large range of variation under different combinations of grinding parameters. Table 7 shows the variance analysis results for the SSD. The *p*-values of the grinding wheel speed, grinding depth, and feed rate were all less than 0.001, indicating that these three process parameters had a significant impact on the ground SSD of the sapphire crystals. The *F*-values corresponding to these three parameters were 88.288, 12.814, and 45.213, respectively. This means that the influence of the grinding wheel speed on the SSD was the most significant, followed by the feed rate and finally the grinding depth. The impact of the grinding parameters on the SSD was consistent with their impact on the *Ra*.

Table 8 shows the range analysis results for the SSD. Factor with larger range values had a more significant impact on the experimental results. The corresponding *R* values for the grinding wheel speed, grinding depth, and feed rate were 5.48, 1.974, and 3.622, respectively, which indicates that within the range of the grinding parameters and under the experimental conditions in this paper, the effect of the grinding wheel speed on the SSD of the ground sapphire crystal was more significant than that of the other two parameters. Among the parameters, the influence of the grinding depth on the SSD was the least significant. The effect of the grinding process on the SSD was consistent with its impact on the *Ra*. The generation of SSD was mainly attributed to the brittle removal of material during grinding, resulting in residual median cracks in the ground subsurface, which appeared as brittle pits on the ground surface. Therefore, in this case, the trend of the change in the surface roughness values was consistent with that for the SSD. The research results for various processing techniques for crystal materials indicate that there is a nonlinear monotonic relationship between the surface roughness and SSD of the processed material when material is removed in the brittle mode during processing [27,28,29]; that is, as the surface roughness increases, the SSD value also increases.

**Figure 6:**
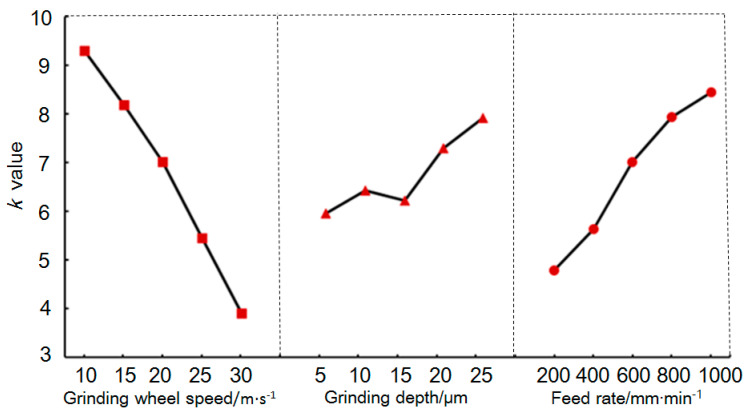
Mean values for various levels of various factors influencing SSD.

Figure 6 shows the average values of the influence at different levels of the three grinding parameters on the SSD. The overall trend was that the SSD values improved with an increase in the grinding wheel speed and a decrease in the grinding depth and feed rate. Compared to the SSD at a grinding depth of 10 μm, when the grinding depth increased to 15 μm, the mean SSD value slightly decreased. A possible reason for this result is that the grinding heat at this grinding depth may have caused some changes in the material properties, such as softening, increased material plasticity, etc., suppressing crack propagation and thus reducing the SSD value. According to the data trend shown in Figure 6, the process parameter combination with which the lowest amount of SSD could be obtained was *A*_1_*B*_5_*C*_1_, which was consistent with the optimal grinding parameter combination found when analyzing the *Ra*.

The SSD needs to be completely removed in the subsequent lapping and polishing process. SSD prediction based on the grinding process parameters is particularly important for improving the surface quality resulting from grinding. Similarly to the development of the model predicting the surface roughness, based on the experimental parameters shown in Table 2 and the corresponding experimental results in Table 6, a power regression mathematical equation for SSD prediction was established, as shown in Equation (2).

SSD = 3.808 *v*_s_^−0.756^*a*_p_^0.148^*v*_w_^0.372^ (2)

Using Equation (2), the amount of SSD under the given grinding parameters can be predicted, which can provide a reference for optimizing the ground surface quality.

### 3.3. Results of Grinding Experiment Using Optimal Process Parameter Combination

**Figure 7:**
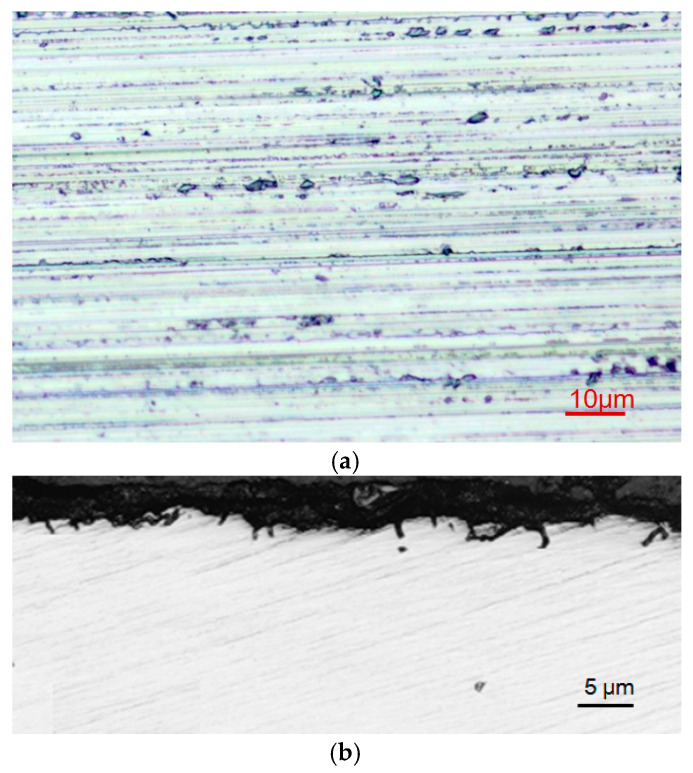
Surface topography (**a**) and subsurface microcrack damage (**b**) of ground sapphire crystal, with grinding wheel speed of *v*_s_ = 30 m·s^−1^, grinding depth of *a*_p_ = 5 μm, and feed rate of *v*_w_ = 200 mm·min^−1^.

Through the range analysis of the orthogonal experimental results for the *Ra* and SSD, it was found that the optimal combination of the grinding process parameters was consistent, with a grinding wheel speed of *v*_s_ = 30 /m·s^−1^, grinding depth of *a*_p_ = 5 μm, and feed rate of *v*_w_ = 200 mm·min^−1^. This set of parameters did not appear in the design of the orthogonal experiment, so a grinding experiment was conducted using this set of parameters. The topography of the ground surface and the subsurface microcrack damage are shown in Figure 7. Under this set of grinding parameters, there were still micro fractured pits on the ground surface due to the brittleness of the material removal during the grinding process. However, the grooves formed through ductile material removal appeared smoother, as shown in Figure 7a. The *Ra* value of the ground surface was 0.326 μm, which was lower than the values of various parameter combinations derived from the orthogonal experiment and consistent with the improvement in the surface morphology. The propagation depth of the microcracks in the ground subsurface decreased, as shown in Figure 7b, resulting in an average SSD of 2.86 μm, which was lower than the values obtained under other grinding parameter combinations in the orthogonal experiment. When aiming to improve the quality of ground surfaces and subsurfaces, this combination of process parameters is suitable.

## 4. Conclusions

A three-factor and five-level orthogonal experiment was conducted on the grinding of single sapphire crystals. Variance and range analysis was conducted on the experimental results by using the *Ra* and SSD as evaluation indicators, and optimized parameter combinations and regression equations for the *Ra* and SSD were established. The conclusions obtained were as follows:

(1) Within the range of the process parameters used in this experiment, the grinding process did not achieve the full ductile removal of the material. Some material was removed in a brittle mode, forming fractured pits on the ground surface, and median crack propagation occurred in the subsurface, forming a subsurface microcrack damage layer. The grinding parameters showed a consistent trend regarding their impact on the surface roughness *Ra* and subsurface microcrack damage depth, which was that the parameter with the greatest impact was the grinding wheel speed, followed by the feed rate and grinding depth.
(2) The values of the *Ra* and SSD improved with an increase in the grinding wheel speed and a decrease in the grinding depth and feed rate. The *Ra* and SSD obtained under the optimized grinding parameter combination were 0.326 μm and 2.86 μm, respectively.
(3) The regression equations for the *Ra* and SSD values established based on the experimental data were *Ra* = 0457*v*_s_^−0.582^*a*_p_^0.114^*v*_w_^0.272^ and SSD = 3.808 *v*_s_^−0.756^*a*_p_^0.148^*v*_w_^0.372^, respectively.

The research results provide an experimental basis for improving the quality of sapphires subjected to grinding and optimizing the process parameters, providing a reference for engineers or researchers in this field.

## Figures and Tables

**Table 1:** Grinding factors and their levels in the orthogonal experiment.

Levels	Factors		
	(A) Grinding Wheel Speed (*v*_s_) /m·s^−1^	(B) Grinding Depth (*a*_p_) /μm	(C) Feed Rate (*v*_w_) /mm·min^−1^
1	30 (A_1_)	25 (B_1_)	200 (C_1_)
2	25 (A_2_)	20 (B_2_)	400 (C_2_)
3	20 (A_3_)	15 (B_3_)	600 (C_3_)
4	15 (A_4_)	10 (B_4_)	800 (C_4_)
5	10 (A_5_)	5 (B_5_)	1000 (C_5_)

**Table 2:** Orthogonal design of grinding parameter combinations.

No.	Parameter Combinations			No.	Parameter Combinations		
1	A_1_	B_1_	C_1_	14	A_3_	B_4_	C_5_
2	A_1_	B_2_	C_3_	15	A_3_	B_5_	C_2_
3	A_1_	B_3_	C_5_	16	A_4_	B_1_	C_3_
4	A_1_	B_4_	C_2_	17	A_4_	B_2_	C_5_
5	A_1_	B_5_	C_4_	18	A_4_	B_3_	C_2_
6	A_2_	B_1_	C_5_	19	A_4_	B_4_	C_4_
7	A_2_	B_2_	C_2_	20	A_4_	B_5_	C_1_
8	A_2_	B_3_	C_4_	21	A_5_	B_1_	C_2_
9	A_2_	B_4_	C_1_	22	A_5_	B_2_	C_4_
10	A_2_	B_5_	C_3_	23	A_5_	B_3_	C_1_
11	A_3_	B_1_	C_4_	24	A_5_	B_4_	C_3_
12	A_3_	B_2_	C_1_	25	A_5_	B_5_	C_5_
13	A_3_	B_3_	C_3_				

**Table 3:** Surface roughness, *Ra*, of ground sapphire crystal wafers.

No.	*Ra* (μm)	No.	*Ra* (μm)	No.	*Ra* (μm)
1	0.354	10	0.517	19	0.775
2	0.442	11	0.812	20	0.515
3	0.501	12	0.555	21	0.836
4	0.349	13	0.669	22	0.939
5	0.458	14	0.729	23	0.634
6	0.727	15	0.527	24	0.788
7	0.451	16	0.848	25	0.871
8	0.611	17	0.942		
9	0.401	18	0.747		

**Table 4:** Variance analysis of ground surface roughness, *Ra*.

Item	Adj MS	Degrees of Freedom	Mean Square	*F*	*p*
Grinding wheel speed (*v*_s_)	0.520	4	0.130	99.139	<0.001
Grinding depth (*a*_p_)	0.057	4	0.014	10.776	<0.001
Feed rate (*v*_w_)	0.224	4	0.056	42.649	<0.001
Error	0.016	12	0.001		
*R*^2^ = 0.981					

**Table 5:** Range analysis for ground surface roughness, *Ra*.

Item	Grinding Wheel Speed (*v*_s_)	Grinding Depth (*a*_p_)	Feed Rate (*v*_w_)
*K*_1_	2.104	3.577	2.459
*K*_2_	2.707	3.329	2.91
*K*_3_	3.292	3.162	3.264
*K*_4_	3.827	3.042	3.595
*K*_5_	4.068	2.888	3.77
*k*_1_	0.4208	0.7154	0.4918
*k*_2_	0.5414	0.6658	0.582
*k*_3_	0.6584	0.6324	0.6528
*k*_4_	0.7654	0.6084	0.719
*k*_5_	0.8136	0.5776	0.754
*R*	0.3928	0.1378	0.2622
Order	1	3	2

**Table 6:** SSD of ground sapphire crystal wafers.

No.	SSD (μm)	No.	SSD (μm)	No.	SSD (μm)
1	3.05	10	5.05	19	8.67
2	4.21	11	9.19	20	5.03
3	4.85	12	5.55	21	9.59
4	3.01	13	7.13	22	11.21
5	4.30	14	7.99	23	6.63
6	7.96	15	5.18	24	8.86
7	4.21	16	9.77	25	10.13
8	6.31	17	11.25		
9	3.61	18	6.17		

**Table 7:** Variance analysis for SSD.

Item	Adj MS	Degrees of Freedom	Mean Square	*F*	*p*
Grinding wheel speed (*v*_s_)	92.442	4	23.110	88.288	<0.001
Grinding depth (*a*_p_)	13.417	4	3.354	12.814	<0.001
Feed rate (*v*_w_)	47.340	4	11.835	45.213	<0.001
Error	3.141	12	0.262		
*R*^2^ = 0.980					

**Table 8:** Range analysis for SSD.

Item	Grinding Wheel Speed (*v*_s_)	Grinding Depth (*a*_p_)	Feed Rate (*v*_w_)
*K*_1_	19.42	39.56	23.87
*K*_2_	27.14	36.43	28.16
*K*_3_	35.04	31.09	35.02
*K*_4_	40.89	32.14	39.68
*K*_5_	46.42	29.69	42.18
*k*_1_	3.884	7.912	4.774
*k*_2_	5.428	7.286	5.632
*k*_3_	7.008	6.218	7.004
*k*_4_	8.178	6.428	7.936
*k*_5_	9.284	5.938	8.436
*R*	5.4	1.974	3.662
Order	1	3	2

## Data Availability

The original contributions presented in this study are included in the article. Further inquiries can be directed to the corresponding author.

## References

[1] Tithy F., Hussain S. (2023). Comprehensive Study of Group III-Nitride Light Emitting Diode Structures Based On Sapphire And Scalmgo4 (0001) Substrate For High Intensity Green Emission. Semicond. Phys. Quant..

[2] Lang L., Saltarelli F., Lacaille G., Rowan S., Hough J., Graumann I., Phillips C., Keller U. (2021). Silicate Bonding Of Sapphire to SESAMs: Adjustable Thermal Lensing for High-Power Lasers. Opt. Express.

[3] Yadav M., Mondal A., Sharma S., Bag A. (2024). Unveiling Thermal Effects on Sn-Doped β-Ga_2_O_3_ Schottky Barrier Diodes on Sapphire for High-Temperature Power Electronics. IEEE Trans. Electron Devices.

[4] Zhao N., Lin Q., Zhu L., Li C., Zhang Z., Yao K., Chen Y., Tian B., Yang P., Jiang Z. (2022). Development of Sapphire Optical Temperature Sensing System Used in Harsh Environment Sensing. IEEE Trans. Instrum. Meas..

[5] Liu Y., Huang H., Wang L., Liao X. (2024). Experimental Study on Normal Force of Cutting Sapphire with Multi-Wire Swing Reciprocating Wire Saw. Diam. Abras. Eng..

[6] Lai Z., Liao X., Yang H., Hu Z., Huang H. (2024). Experimental Study on the Formation Mechanism of Saw Marks in Wire Sawing. Int. J. Mech. Sci..

[7] Zhu Z., Gao Y., Shi Z., Zhang X. (2023). Study on Surface Characteristics Of As-Sawn Sapphire Crystal Wafer Considering Diamond Saw Wire Wear. Wear.

[8] Gupta A., Chen C., Hsu H. (2019). Study on Diamond Wire Wear, Surface Quality, and Subsurface Damage during Multi-Wire Slicing of C-Plane Sapphire Wafer. Int. J. Adv. Manuf. Technol..

[9] Zhu Z., Gao Y., Zhang X. (2023). Study on Subsurface Microcrack Damage Depth of Diamond Wire as-Sawn Sapphire Crystal Wafers. Eng. Fract. Mech..

[10] Wang S., Tie G., Shi F., Tian Y., Yang X. (2024). Effect of Different Grinding Strategies on Subsequent Polishing Processes of Sapphire. J. Manuf. Process..

[11] Xu Y., Sun J., Zhan H., Fu B., Zhan Y., Zheng T. (2023). Performance of Thermal Field-assisted Precision Lapping for Single Crystal Sapphire Wafers. Diam. Abras. Eng..

[12] Yang H., Wang Y., Liang Z., Su Y., Xu Z., Guo R., Wang B. (2018). Research on Sapphire Surface MicroStructure Processed by Diamond Grinding Wheel. New Technol. New Process.

[13] Wang S., Zhao Q., Yang X. (2022). Surface and Subsurface Microscopic Characteristics in Sapphire Ultra-precision Grinding. Tribol. Int..

[14] Wang X., Zheng W., Bao X., Zhao Q. (2025). Anisotropy Monitoring of Ultra-precision Grinding Force and Acoustic Emission Signal of Monocrystalline Sapphire. Measurement.

[15] Hu Z., Shao M., Guo J., Huang S., Xu X. (2017). Comparison of Grinding Characteristics of Different Crystal Surfaces for Sapphire. Opt. Precis. Eng..

[16] Cheng J., Yu T., Wu J., Jin Y. (2018). Experimental Study on “Ductile-brittle” Transition in Micro-grinding of Single Crystal Sapphire. Int. J. Adv. Manuf. Technol..

[17] Wang Q., Liang Z., Wang X., Zhao W., Wu Y., Zhou T. (2015). Fractal Analysis of Surface Topography in Ground Monocrystal Sapphire. Appl. Surf. Sci..

[18] Zhou F., Xu J., Zhang W., Yu H. (2024). Research on Quality of Bottom Surface of Sapphire Micro-grooves Ground by Structured Grinding Wheels. J. Hunan Univ. Nat. Sci..

[19] Zhou F., Xu J., Zhang Y., Zhang W., Zhao B., Yu H. (2024). Numerical simulation and experimental study of micro-structured surface of sapphire ground by structured grinding wheel. Precis. Eng..

[20] Wang S., Zhao Q., Zhao Q., Zhou M. (2025). Investigate on Surface/Subsurface Damage Mechanisms and Manufacturability of Ultra-smooth Surface in Ultra-precision Ductile Grinding of Sapphire Optics. J. Mater. Res. Technol..

[21] Sun G., Zhang W., Wang J., Ding J., Wang B., Shi F. (2024). Research on Subsurface Damage Mechanism and Suppression Method of Ultrasonic Vibration-assisted Grinding of Sapphire Components under Extreme Service Environment. Int. J. Adv. Manuf. Technol..

[22] Xiao H., Yin S., Zhou P., Wu H. (2025). Prediction of Grinding Parameters Based on Specific Surface Integrity of Hard-brittle Materials. J. Manual. Process..

[23] Wasmer K., Pochon P., Sage D., Giovanola J. (2017). Parametric Experimental Study and Design of Experiment Modelling of Sapphire Grinding. J. Clean. Prod..

[24] Peng C., Li G., Zhang X., Gao Y. (2025). Process Parameters Analysis in Diamond Wire Saw Cutting NdFeB Magnet. Materials.

[25] Hasçelik A., Aslantas K., Yalçın B. (2025). Optimization of Cutting Parameters to Minimize Wall Deformation in Micro-Milling of Thin-Wall Geometries. Micromachines.

[26] Huang X., Wang T., Hu S., Yao T., Miao R., Kang Q., Zhang Y. (2022). Parameter Optimization of Laser Based on Orthogonal Experiment and Response Surface Method. Laser Optoelectron. Prog..

[27] Li S., Wang Z., Wu Y. (2008). Relationship between Subsurface Damage and Surface Roughness of Optical Materials in Grinding and Lapping Processes. J. Mater. Process. Technol..

[28] Zhu Z., Gao Y., Shi Z. (2024). Relationship between Surface Roughness and Subsurface Crack Damage Depth of Sapphire Crystals Cut by Diamond Wire Saw Based on Slicing Experiments. Int. J. Adv. Manuf. Technol..

[29] Wang K., Gao Y., Yang C. (2024). Prediction of Subsurface Microcrack Damage Depth Based on Surface Roughness in Diamond Wire Sawing of Monocrystalline Silicon. Materials.

